# The Impact of Cigarette Excise Tax Increases on Regular Drinking Behavior: Evidence from China

**DOI:** 10.3390/ijerph17093327

**Published:** 2020-05-11

**Authors:** Zili Zhang, Rong Zheng

**Affiliations:** 1 School of International Trade and Economics, University of International Business and Economics, Beijing 100029, China; 201700130006@uibe.edu.cn; 2 World Health Organization Collaborating Center on Tobacco and Economics, 10 Huixin East Street, Chaoyang District, Beijing 100029, China

**Keywords:** cigarette, tax, drinking behavior

## Abstract

(1) *Background:* Many studies have shown that increasing taxation on cigarettes does play a role in tobacco control, but few studies have focused on whether increasing cigarette excise taxes significantly affects alcohol consumption. In this article, we aim to examine the effects of China’s 2015 increase in the cigarette excise tax on residents’ regular drinking behavior. (2) *Methods:* Using survey data from China Family Panel Studies (CFPS), we performed a panel logit regression analysis to model the relationship between the cigarette excise tax and regular drinking behavior. The Propensity Score Matching with Difference-in-Differences (PSM-DID) approach was adopted to determine the extent to which the cigarette excise tax affected residents’ drinking behavior. To test whether the cigarette excise tax could change regular drinking behavior by decreasing daily smoking quantity, we used an interaction term model. (3) *Results:* China’s 2015 increase in the cigarette excise tax had a significant negative effect on the probability of regular alcohol consumption among smokers, and the cigarette excise tax worked by reducing the average daily smoking of smokers. We also found that the regular drinking behavior of male smokers was more deeply affected by the increased cigarette excise tax than females. (4) *Conclusions:* Our research results not only give a deeper understanding of the impact of the cigarette excise tax, but also provide an important reference with which to guide future decisions concerning excise taxes imposed on cigarettes.

## 1. Introduction

Tobacco and alcohol are among the top causes of preventable deaths [1]. Smoking is associated with lung disease, cancers, and cardiovascular disease [2]. Alcohol is associated with chronic liver disease, cancers, cardiovascular disease, and fetal alcohol syndrome. When combined, tobacco and alcohol dramatically increase the risk of certain cancers [3]. Not only does tobacco and alcohol consumption pose health hazards to individuals, it also increases the medical burden on society to a greater extent. Studies have shown that alcohol consumption costs the United States nearly $185 billion annually in direct and indirect costs, while the health costs of smoking are nearly $158 billion [4,5]. Although both tobacco and alcohol are harmful, tobacco is subject to more government restrictions than alcohol in China. To reduce the harm of tobacco, the Chinese government raised the cigarette excise tax on 1 May 2015, and this policy did result in lower cigarette consumption [6]. As the co-use of tobacco and alcohol has always been common in China, we found an interesting phenomenon from the China Family Panel Studies (CFPS) data: compared with that of non-smokers, the regular drinking rate of smokers decreased significantly. Therefore, in order to test whether the cigarette excise tax caused this phenomenon, this article aims to examine the effects of the cigarette excise tax on regular drinking behavior.

To date, there is no research on the cross-effects of smoking and drinking policies in Mainland China. The research results of other countries and regions are not consistent. Many of these studies have identified cigarettes and alcohol as complementary products and suggested that regulation of one item may lead to a decline in demand for the other [7,8,9,10,11,12]. For example, Melissa J. Krauss et al. (2014) analyzed U.S. state-level data from 1980 to 2009 and found that tax increases led to higher cigarette prices, which led to a reduction in both cigarette and alcohol consumption [9]. Kelly C. Young-Wolff et al. (2014) examined data from a longitudinal study in the United States and found that smokers’ typical alcohol consumption and binge drinking were significantly reduced compared to consumption in states without increased tobacco taxes [12]. Jie-Min Lee (2007) found that cigarettes, betel nuts, and alcoholic beverages had complementary relationships in Taiwan. The health tax on cigarettes would reduce cigarette consumption, betel nut consumption, and alcohol consumption at the same time [10]. Pierpaolo Pierani and Silvia Tiezzi (2009) used an analysis of time-series data on per capita expenditures and prices from 1960 to 2002 and found that raising alcohol taxes effectively reduced alcohol and tobacco consumption. However, other studies had different conclusions [11]. Rajeev K. Goel and Mathew J. Morey (1995) suggested that cigarettes and liquor were substitutes in consumption and that raising cigarette taxes would increase liquor consumption. The complete dataset they used included nearly 800 observations organized by year and by state between 1959 and 1982 in the United States [13]. Sandra L. Decker and Amy E. Schwartz (2000) and Gabriel A. Picone et al. (2004) also found an increase in cigarette price/tax associated with an increase in alcohol use [14,15].

Our research is the first article in China dedicated to the impact of the cigarette excise tax on regular drinking behavior. We focus on whether raising the cigarette excise tax affects the regular drinking behavior of smokers and the mechanisms of influence. China’s 2015 cigarette excise tax reform provides an opportunity for our research. To address the endogeneity problem posed by sample selection bias and omitted variables, the Propensity Score Matching with Difference-in-Differences (PSM-DID) method is used. Since the explanatory variables are 0, 1 variables, we use the panel logit fixed effects model. For further mechanism testing, we use the interaction term model. Our results show that increasing the cigarette excise tax did reduce the probability of regular drinking among smokers and that the reduction in average daily cigarette consumption is a channel through which the cigarette excise tax affects regular drinking behavior.

The rest of the paper is structured as follows: Section 2 introduces the background, survey data, and our models. Section 3 shows the regression results of the benchmark model and the extended model. The discussion is contained in Section 4. The last section concludes the paper.

## 2. Materials and Methods 

### 2.1. Background

**Figure 1:**
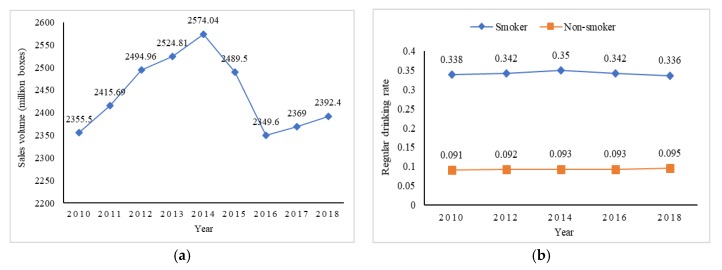
(**a**) Cigarette sales volume in China (2010–2018). (**b**) Regular drinking rate of smokers and non-smokers.

China’s cigarette excise taxes changed only once between 2010 and 2018. On 1 May 2015, the Chinese government increased the ad valorem excise tax rate for cigarette wholesale from 5% to 11%. As we know, the specific behavior and social responsibility of corporations can have a greater impact on policy transmission [16]. To cope with tax changes, the China National Tobacco Corporation increased the wholesale prices of all domestic cigarettes and imported cigarettes by 6%, and the retail price of cigarettes also increased accordingly [6]. Cigarette sales volume data from the China Tobacco Almanac are shown in Figure 1a. Due to the cigarette excise tax increases, there had been a markedly different trend in cigarette sales of China National Tobacco Corporation since 2015.

To set the stage for our empirical work, it is also important to look at changes in regular drinking rates. By observing the data of China Family Panel Studies, we show the different regular drinking rates between smokers and non-smokers in Figure 1b. There are two important features to note at this point. The first is that the regular drinking rate of smokers was much higher than that of non-smokers. The second point to note is that the regular drinking rate of smokers after the increase of cigarette consumption tax declined significantly, while the non-smokers’ regular drinking rate did not change significantly. The obvious decline in smokers ’regular drinking behaviors after 2015 provided a realistic basis for our empirical research.

### 2.2. Data

This study used China Family Panel Studies (CFPS) data. The survey was initiated by the Institute of Social Science Survey (ISSS) at Peking University and was jointly conducted by the ISSS and the University of Michigan Research Center. Given the stratified multistage sampling design, the population in the sample area accounted for 94.5% of the total population. The survey data mainly included data on communities, families, family members, adults, and children. In this article, we used CFPS 2010, 2012, 2014, 2016, and 2018 national-level adult survey data for analysis. All analyses were conducted in Stata MP 15.0. The total number of 5 year observations in the initial sample was 169,852. We adjusted the sample and kept only the observations that existed in the five surveys. The adjusted effective sample consisted of 71,570 people, including 33,595 men and 37,975 women. The number of smokers in the treatment group was 20,686, and the number of non-smokers in the control group was 50,884. The descriptive statistics of the data are shown in Table 1.

The explanatory variable in this article was whether the respondent currently drinks alcohol regularly. The question in the questionnaire was: “Do you drink more than 3 times a week?” If the respondent regularly drank more than three times a week, the value of Regulardrinker was 1; otherwise, the value was 0.

Currentsmoker was a main explanatory variable in this article. The questionnaire asked “if there was smoking last month”; if the answer was yes between 2010 and 2018, then the Currentsmoker value was 1; if the answer was no between 2010 and 2018, the value was 0. In order to keep the individuals in the control group and the experimental group unchanged during the use of the DID method, we deleted the sample of respondents whose answers changed. The variable Tax was a dummy variable that indicated whether it was before or after the cigarette tax increase. The value of Tax before 2015 was 0, and the value of Tax after 2015 was 1. For the mechanism of analysis, we used Daysmokenum as an explanatory variable. Daysmokenum indicated the average daily smoking reported by respondents, with a value of 0 for nonsmokers. The relevant question in the questionnaire was “How many cigarettes do you currently smoke per day on average?”.

With regard to the selection of control variables, this paper drew on previous research literature [9,17]. The main control variables included Smokefree, Smokeyear, Age, Urban, Marriage, Lnincome, Education, and Chronic. Smokefree represented whether the interviewee was affected by Smoke-Free Air laws (SFA laws). The main cities in China that have implemented smoke-free legislation are Shanghai (2010), Shenzhen (2014), Beijing (2015), etc. Since SFA laws are only enforced at the urban level, the local rural population is virtually unaffected. Thus, if a city adopted smoke-free legislation, local urban respondents were considered to have been affected, with Smokefree taking a value of 1 and 0 otherwise. Smokeyear indicated the duration of smoking. Age was a continuous variable, and the age of respondents in our sample was 16 and over. Urban was a dummy variable. The values were 1 for city and 0 for rural. Marriage was also a dummy variable. If the respondent was married, the value was 1; otherwise, it was 0. Education level was a categorical variable, with the value of 1 for no schooling, the value of 2 for primary school education, the value of 3 for junior high school education, the value of 4 for high school education, the value of 5 for junior college education, the value of 6 for college education, and the value of 7 for graduate education. Lnincome indicated the logarithm of respondents’ income. Chronic referred to whether the respondent had a chronic disease within the last six months. If the answer was yes, the value was 1; otherwise, the value was 0.

### 2.3. Methods

#### 2.3.1. Theoretical Analysis of the Model Setting

This article considered smokers as a treatment group and nonsmokers as a control group, using the Propensity Score Matching with Difference-in-Differences (PSM-DID) method to isolate the net effect of increasing tobacco taxes on regular drinking behavior. On the one hand, the increase in China’s cigarette excise taxes was promoted by legislature in order to adapt to China’s economic and social development. For an individual, it was an event beyond his or her control, so it could be regarded as an external shock. The exogenous shock of the policy provided the basis for our assessment using the Difference-in-Differences (DID) method. On the other hand, the Propensity Score Matching (PSM) prior to DID estimation could better reduce selectivity bias, and the PSM-DID method could not only take advantage of the above-mentioned multivariate method, but also effectively control differences in observable characteristics between the participant and control groups through the PSM method. This paper applied this method in two main steps: the first step was to match the propensity values of the treatment and control groups with the 2010 data and to remove data that were not successfully matched. The second step was to form a balanced panel with the matched data and data from other years and then use that data and the DID method for panel regression analysis. 

The policy effects of interest in this paper were the Average Treatment effect on the Treated (ATT), i.e., the change in regular drinking behavior of individuals in the smoking group as a result of higher tobacco taxes. Formally, the ATT can be expressed as follows:

(1) $$ATT=E\left(Y_{i,post}^{P}-Y_{i,pre}^{P}|D_{i}=1\right)-E\left(Y_{i,post}^{NP}-Y_{i,pre}^{NP}|D_{i}=1\right)$$

$Y_{i,pre}^{P}$ and $Y_{i,post}^{P}$ represent the potential outcomes of drinking behavior before and after the tax increase if individual *i* is a current smoker, respectively; $Y_{i,pre}^{NP}$ and $Y_{i,post}^{NP}$ represent the potential outcomes of drinking behavior before and after the tax increase if individual *i* is not a current smoker, respectively; $D_{i}$ is a dummy variable, and $D_{i}=1$ indicates that individual *i* is a current smoker and vice versa. When model estimation was carried out, the simple use of $E(Y_{i,post}^{NP}-Y_{i,pre}^{NP}|D_{i}=0)$ a proxy for unobservable $E(Y_{i,post}^{NP}-Y_{i,pre}^{NP}|D_{i}=1)$ led to selective bias. Heckman et al. (1998) demonstrated that ATT can be estimated based on the following equation [18]:

(2) $$ATT=E_{P\left(X_{i}\right)|D_{i}=1}\left\{E\left(Y_{i,post}^{P}-Y_{i,pre}^{P}|P\left(X_{i}\right),D_{i}=1\right)-E\left(Y_{i,post}^{NP}-Y_{i,pre}^{NP}|P\left(X_{i}\right),D_{i}=0\right)\right\}$$

$P\left(X_{i}\right)=\mathrm{Pr}(D_{i}=1|X_{i})$ is the propensity score function, i.e., the probability that an individual *i* smokes given a set of observable characteristics $X_{i}$. In estimating the propensity score function, we chose the logit model: the explanatory variable was $D_{i}$, and the explanatory variables were those that affected both current smoking behavior and frequent drinking behavior, such as gender, age, education level, and income. After estimating the propensity score for each individual, the samples could be matched accordingly. Individuals falling within the common support propensity score range were selected, and each smoker could be matched with one or more propensity score that was close enough to his/her non-smoker counterpart. This article used the nearest neighbor matching method. Since the ratio of processing group to control group data was 1:2.46, we used a matching ratio of 1:2 with a matching error range of 0.01. Ultimately, each individual in the treatment group matched two more similar individuals in the control groups within 1% of the difference in propensity values. Data with no successful matches would be removed before the next DID regression analysis.

#### 2.3.2. Baseline Model

Based on Equations (1) and (2), this paper estimated the average treatment effect of higher cigarette excise taxes on the regular drinking behavior of smokers. DID analysis of the data after processing using the PSM method was performed to mitigate sample selection bias and the impact of missing variables or unobserved factors on the accuracy of the results. Referring to the research of Thorsten Beck et al. (2010) [19] and Marianne Bertrand et al. (2004) [20], we established the following function:

(3) $$Regulardrinker_{it}=\alpha_{0}+\alpha_{i}+\alpha_{t}+\alpha_{1}Tax_{t}\times Currentsmoker_{i}+\alpha_{2}X_{it}+\varepsilon_{it}$$

The dependent variable $Regulardrinker_{it}$ represents the drinking behavior of individual *i* during year *t*. The explanatory variable $Currentsmoker_{i}$ indicates whether respondent *i* is a current smoker between 2010 and 2018. $X_{it}$ in the equation represents a number of individual characteristic variables, including age, living in urban or rural areas, marital status, income, and so on. $\alpha_{0}$ is the constant term. $\alpha_{t}$ is the time fixed effect, which was used to control unknown effects that changed over time, but not with individuals. $\alpha_{i}$ is the individual fixed effect, used to capture differences (e.g., gender, ethnicity, and birthplace) between individuals that did not change over time. $\varepsilon_{it}$ is the residual term, which represented other unobserved factors affecting the smoking behavior of individuals.

#### 2.3.3. Model for the Mechanism Test

To test the hypothesis that reducing daily average cigarette consumption was how cigarette excise taxes affected residents’ drinking, we set up Equation (2):

(4) $$Regulardrinker_{it}=\alpha_{0}+\alpha_{1}Tax_{t}+\alpha_{2}Daysmokenum_{it}+\alpha_{3}Tax_{t}\times Daysmokenum_{it}+\alpha_{4}X_{it}+\alpha_{i}+\alpha_{t}+\varepsilon_{it}$$

In Equation (4), we include an interaction term between $Tax_{t}$ and $Daysmokenum_{it}$ to examine the effect of the cigarette excise tax on regular drinking behavior. What we were primarily concerned with in our analysis was the coefficient of $Tax_{t}\times Daysmokenum_{it}$. As in Equation (3), we also controlled for time fixed effects and individual fixed effects.

## 3. Results

### 3.1. t-Test for Data before and after the PSM 

The hypothesis that there was no significant difference in covariates between the smoking and non-smoking groups after the PSM method matching underlay the application of the PSM-DID method. To test whether PSM method matching was effective at reducing the difference in covariates between the smoking and non-smoking groups, we performed a *t*-test for the covariates in the model. The test results are shown in Table 2. Compared with the pre-match (UnMatched) data, the standardized deviations of the characteristic variables between both smoking and non-smoking groups were substantially reduced in the post-match (Matched) data. For example, before matching, there was a significant gender difference between the smoking and non-smoking groups, with 96.5% of men in the smoking group and 24.7% of men in the non-smoking group. After matching, the proportion of males in the non-smoking group was 96.5%, consistent with the smoking group, with a 100% reduction in the gender difference between the two groups. Similarly, differences in indicators such as age, education, and income between the smoking and non-smoking groups were significantly reduced after matching. The *t*-test results for each indicator were not significant, and the original hypothesis that the covariates were not significantly different between the smoking and non-smoking groups was not rejected. Overall, the mean standardized bias of the variables between the two matched groups was reduced from 36.4% to 1.7%, essentially eliminating the selection bias caused by grouping based on smoking or not.

### 3.2. Estimation Results of PSM-DID

Table 3 shows the panel logit regression results using the PSM-DID method. The regression results from Column (1) to Column (5) show the stepwise addition of control variables. Column 5 reports regression results for the inclusion of all control variables, time fixed effects, and individual fixed effects. The coefficient of the interaction term is significant in Column (5), indicating that the probability of a decrease in regular drinking behavior among smokers was 2.111 = [1/exp(−0.747)] times that of non-smokers affected by taxation. The regression coefficients of the control variables indicated that SFA laws had no significant effect on regular drinking behavior. The duration of smoking had a positive effect on regular drinking behavior. The likelihood of residents drinking regularly increased with age and then decreased. There was also a positive correlation between income and regular drinking behavior. Moreover, marriage had a significant negative effect on regular drinking behavior, and people with chronic diseases in the past six months had a significantly lower probability of regular drinking.

### 3.3. Mechanism Analysis

Based on the previous conclusions of the PSM-DID method, we believed that the cigarette excise tax had a significant effect on the regular drinking behavior of smokers. To test whether the cigarette excise tax could change regular drinking behavior by reducing daily smoking quantity, we used an interaction term model, Equation (4), as shown in Section 2. We used full-sample data for regression in this section, not data after PSM. Table 4 reports the panel logit regression results of the mechanism analysis. The coefficient of Daysmokenum×Tax was significantly negative when controlling for other variables, which indicated that the cigarette excise tax influenced smokers’ daily drinking behavior by affecting their daily consumption of cigarettes. Specifically, for smokers who smoked one cigarette a day on average, the probability of a decline in regular drinking behavior would be 1.016 = [1/exp(−0.0162)] times higher for non-smokers, influenced by taxation. For smokers who smoked ten cigarettes a day on average, the probability of a decrease in regular drinking behavior would be 1.176 = [1/exp(−0.0162 × 10)] times that of non-smokers. In the logit model, the effects of taxation and average daily smoking on frequent drinking behavior were not linear. Overall, smokers who smoked more per day were more likely to experience a decline in their regular drinking behavior as a result of the tax increase. The coefficients of the other control variables were largely consistent with those of the benchmark model regression results in Table 3, as explained above.

### 3.4. Heterogeneity Analysis

Alcohol and tobacco use vary according to factors such as gender and age. Men’s use is usually higher than women’s use [21]. Therefore, to study the heterogeneous impact of the cigarette tax on the regular drinking behavior of different populations, we analyzed the samples by gender and three different age groups. Since this part was analyzed using the PSM-DID method, we used the data after the PSM match.

Table 5 shows the regression results of the fixed effects logit model by gender and age group. The coefficient of Currentsmoker×Tax in Column (1) is significantly negative, indicating that the probability of a decrease in regular drinking behavior among male smokers was 2.182 = [1/exp(−0.780)] times that of male non-smokers affected by the cigarette excise tax. The interaction term coefficient in Column (2) did not significantly indicate that increasing the cigarette tax had no significant effect on the regular drinking behavior of female smokers. The regression results in Columns (3)–(5) showed that the 2015 increase in the cigarette excise tax reduced the probability of regular drinking behavior in people aged 34–55. However, the effect on regular drinking behavior was not significant in people aged 16–34 and over 55 years.

## 4. Discussion

The main findings of this study confirmed that the increase in the cigarette excise tax had a significant negative impact on smokers’ regular drinking behavior. Specifically, our regression results using the PSM-DID method showed that smokers were two times more likely than non-smokers to experience a decrease in regular drinking behavior due to the cigarette excise tax. Most of the existing research focused on the impact of cigarette excise taxes on alcohol consumption [9,10,15]. Our research on the regular drinking behavior of smokers was an innovative supplement to the existing literature.

SFA laws in cities may have an impact on the smoking and drinking behaviors of local residents. To avoid the estimation bias caused by the omission of this variable, we used SFA laws in cities as a control variable in the regression analysis. The results in Table 3 and Table 4 show that the effect of SFA laws on residents’ regular drinking behavior was not significant. This was not consistent with the conclusion of Melissa J. Krauss et al. (2014), who used U.S. state-level data to conclude that smoke-free legislation had a significant negative impact on drinking behavior [9]. In our opinion, the main reason was that China’s smoke-free legislation has been implemented for a relatively short time, with relatively weak enforcement. In fact, Lin et al.’s (2019) study on smoke-free legislation in Chinese cities also pointed out that although the number of Chinese cities undertaking smoke-free legislation was increasing, the scope of legislation varied widely. As of 2019, only nine cities have banned smoking in all indoor workplaces and common public places [22].

For the first time, we verified that daily cigarette consumption was one of the channels through which cigarette excise taxes affected the regular drinking behavior of smokers. Many previous studies proved that tobacco and alcohol were complementary products [7,8,9,10,11,12]. Smokers are more likely to drink alcohol [23], and smokers are four times more likely to rely on alcohol than nonsmokers [24]. Therefore, we hypothesized that increasing the cigarette excise tax would reduce regular drinking among smokers through a decline in cigarette consumption. The results of the mechanism analysis in Table 4 confirmed this hypothesis. We also found that smokers with higher average daily cigarette consumption were more affected by the cigarette excise tax increase. The discovery of this mechanism may mean that measures that help smokers cut down on their daily smoking habit would also reduce their regular drinking behavior.

Since regular smoking prevalence in China varied significantly by gender (males 52.1%, females 2.7% in 2015) [25], we analyzed the male and female samples separately. When analyzing by gender, we found that the cigarette excise tax had a significant effect on regular drinking behavior in men, but not in women. In our opinion, gender disparities in smoking and drinking may be attributable to historically social factors. More normative constraints are imposed on women than men [26,27]. Fewer restrictions mean that men are more self-directed [28]. Therefore, the effect of the cigarette excise tax on men’s smoking and regular drinking behavior was not likely to be influenced by other factors, and the effect of the cigarette excise tax on men was more significant than that for women.

Analysis by age group showed that the cigarette excise tax reduced the regular drinking behavior of smokers aged 34–55, while smokers under the age of 34 and those over 55 were less affected by the cigarette excise tax. There were two main reasons for this result: On the one hand, young people aged 16–34 were in an upward phase of social activity, and increased social demand strengthened their probability of passive drinking, thereby weakening the effect of the cigarette excise tax. On the other hand, compared with older smokers who had been smoking longer, young smokers had a relatively low degree of tobacco addiction [29,30]. Increasing tobacco taxes could lead to a greater decline in their average daily smoking because of the link between smoking and alcohol consumption [31,32]. Our conclusions were different from those of Deborah, L. McLellan et al. (2012). In their view, raising cigarette prices though tobacco taxes would lead to an increase in alcohol consumption among people aged 21–29 and those aged 65 and older [33]. The data they used were data from the 2001–2006 survey in the United States. These differences in conclusions may be caused by different national conditions.

## 5. Conclusions

This paper added to the existing literature on the relationship between the cigarette excise tax and smoker’s regular drink behavior using Chinese five year panel data. The results of the PSM-DID method showed that increasing the cigarette excise tax clearly led to a decline in regular drinking behavior among smokers. Such results provide an important reference with which to guide future decisions concerning health taxes imposed on cigarettes.

Another major finding of this paper was that the reduction in daily smoking was a channel through which higher tobacco taxes influenced regular drinking behavior among smokers. This conclusion implied that other measures that lead to a decrease in average cigarette smoking, such as flat-price packaging of cigarettes and bans on tobacco advertising, may also lead to a decrease in alcohol consumption among smokers. Therefore, examining the specific effects of other tobacco control policies on residents’ drinking behavior may also be an interesting research direction.

In addition, the consequences of cigarette tax increase for alcohol drinking behaviors among Chinese smokers indicated potential collaboration and coordination on policy-making regarding tobacco control and alcohol prevention. China is on its comprehensive and ambitious way towards the Healthy China 2030 Initiative, to reach the targets of both tobacco control and alcohol prevention set by Heathy China 2030, an economic modeling is needed by integrating the correlation between smoking and alcoholic drinking so as to arrive at an optimal excise tax for the purpose of promoting health while maximizing the government’s revenue.

## Figures and Tables

**Table 1:** Summary statistics.

Variables	N	Mean	Std. Dev.	Min	Max
Regulardrinker	71,570	0.164	0.370	0	1
Currentsmoker	71,570	0.289	0.453	0	1
Tax	71,570	0.4	0.490	0	1
Daysmokenum	71,402	4.739	9.248	0	100
Smokefree	71,570	0.058	0.233	0	1
Smokeyear	71,570	9.017	15.518	0	76
Male	71,570	0.469	0.499	0	1
Age	71,570	49.752	13.769	16	94
Urban	71,216	0.456	0.498	0	1
Marriage	71,569	0.884	0.320	0	1
Lnincome	30,590	8.902	1.827	0	14.4
Edu	71,549	2.525	1.281	1	7
Chronic	71,557	0.177	0.382	0	1

**Table 2:** Balancing tests from neighbor matching.

**Part A. Test the Balancing Property for Each Observed Covariate**							
**Variable**	**Unmatched**	**Mean**		**Bias%**		***t*** **-Test**	
	**Matched**	**Smoker**	**Non-Smoker**	**Bias%**	**Reductbias%**	***t*** **-Value**	***p*** **-Value**
Gender	U	0.965	0.247	216.9		80.47	0.000
	M	0.965	0.965	0.0	100.0	−0.00	1.000
Age	U	45.984	45.392	4.8		2.00	0.045
	M	45.987	46.116	−1.0	78.2	−0.38	0.706
Age2	U	2259.1	2223.1	3.2		1.32	0.187
	M	2259.8	2272.1	−1.1	65.9	−0.39	0.695
Edu	U	2.619	2.537	6.7		2.76	0.006
	M	2.619	2.631	−1.0	85.3	−0.36	0.715
Lnincome	U	8.925	8.428	33.9		13.73	0.000
	M	8.926	8.941	−1.0	97.0	−0.40	0.69
Urban	U	0.406	0.454	−9.7		−4.13	0.000
	M	0.406	0.420	−2.9	70.0	−1.04	0.300
Marriage	U	0.889	7.601	−5.2		−2.26	0.024
	M	0.889	7.671	1.2	77.2	0.40	0.686
Chronic	U	0.118	0.156	−10.9		−4.51	0.000
	M	0.118	0.100	5.4	50.6	2.09	0.037
**Part B. Test the overall balance**							
Sample	LR χ2		*p* > χ^2^		Meanbias	Medbias	
Unmatched	4482.79		0.000		36.4	8.2	
Matched	6.27		0.617		1.7	1.1	

Note: Unmatched (U) is the data before the smoking and non-smoking groups were matched, and Matched(M) is the data after the match.

**Table 3:** Regular drinking behavior responses to the cigarette tax.

Variable	(1)	(2)	(3)	(4)	(5)
	Regulardrinker	Regulardrinker	Regulardrinker	Regulardrinker	Regulardrinker
Tax × Currentsmoker	0.133 **	−0.00466	−0.754 **	−0.746 **	−0.747 **
	(0.0672)	(0.200)	(0.354)	(0.355)	(0.359)
Smokeyear		0.0478	0.0943 *	0.0903 *	0.0861 *
		(0.0345)	(0.0493)	(0.0495)	(0.0501)
Smokefree		0.720	1.081 *	1.059	1.032
		(0.455)	(0.654)	(0.654)	(0.661)
Lnincome			0.0816 ***	0.0582 **	0.0616 **
			(0.0287)	(0.0306)	(0.0310)
Age				0.0218	0.0579
				(0.437)	(0.443)
Age2				−0.00170 ***	−0.00198 ***
				(0.000601)	(0.000631)
Edu					0.0389
					(0.160)
Urban					−0.409
					(0.266)
Marriage					−0.484 *
					(0.263)
Chronic					−0.476 ***
					(0.147)
Individual Fixed	N	Y	Y	Y	Y
Time Fixed	N	Y	Y	Y	Y
N	15815	6565	2549	2549	2530
LR chi^2^	3.92	16.06	21.54	29.77	46.20
Prob > chi^2^	0.0478	0.0246	0.0058	0.0009	0.0000

Notes: Standard errors in parentheses; ***, **, and * indicate significance at the 1, 5, and 10% levels; individual and time fixed effects were controlled.

**Table 4:** Regular drinking behavior responses to the cigarette tax and Daysmokenum.

Variable	(1)	(2)	(3)	(4)	(5)
	Regulardrinker	Regulardrinker	Regulardrinker	Regulardrinker	Regulardrinker
Daysmokenum	0.0904 ***	0.0364 ***	0.0400 ***	0.0390 ***	0.0394 ***
	(0.0027)	(0.0033)	(0.0052)	(0.0053)	(0.0054)
Tax	−0.0443	−0.0388	0.0689	0.937	0.443
	(0.0380)	(0.0661)	(0.108)	(1.416)	(1.469)
Daysmokenum × Tax	0.0058 *	0.0041	−0.0163 **	−0.0154 **	−0.0162 **
	(0.0031)	(0.0036)	(0.00667)	(0.00670)	(0.0068)
Smokeyear		0.00362	0.0300	0.0301	0.0296
		(0.0129)	(0.0206)	(0.0207)	(0.0210)
Smokefree		0.164	0.301	0.302	0.312
		(0.268)	(0.424)	(0.424)	(0.426)
Lnincome			0.0738 ***	0.0570 **	0.0583 **
			(0.0244)	(0.0255)	(0.0257)
Age				−0.0266	0.0607
				(0.181)	(0.187)
Age2				−0.000911 **	−0.00120 ***
				(0.000389)	(0.000411)
Edu					−0.00901
					(0.100)
Urban					−0.418 **
					(0.176)
Marriage					−0.504 ***
					(0.174)
Chronic					−0.388 ***
					(0.0924)
Individual Fixed	N	Y	Y	Y	Y
Time Fixed	N	Y	Y	Y	Y
N	71570	18390	6083	6083	5984
LR chi^2^	143	90.01	89.25	95.15	125.01
Prob > chi^2^	0.0000	0.0000	0.0000	0.0000	0.0000

Notes: Standard errors in parentheses; ***, **, and * indicate significance at the 1, 5, and 10% levels; individual and time fixed effects were also controlled.

**Table 5:** Regular drinking behavior responses to the cigarette tax by gender and age groups.

Variable	Gender		Age Groups		
	(1)	(2)	(3)	(4)	(5)
	Male	Female	16–34	35–54	55–
Currentsmoker × Tax	−0.780 **	0.119	−0.0808	−1.143 ***	−0.644
	(0.3630)	(0.0869)	(1.000)	(0.024)	(0.744)
Control Variables	Y	Y	Y	Y	Y
Individual Fixed	Y	Y	Y	Y	Y
Time Fixed	Y	Y	Y	Y	Y
N	2394	135	398	1065	594
Wald chi^2^	47.56	5.41	18.54	37.72	25.10
Prob > chi^2^	0.0000	0.0914	0.1832	0.0051	0.0336

Notes: Standard errors in parentheses; *** and ** indicate significance at the 1% and 5% levels; other control variables and individual and time fixed effects were controlled.
